# Supplementary material for: Associations Between 10-Year Physical Performance and Activities of Daily Living Trajectories and Physical Behaviors in Older Adults
Source: Int J Environ Res Public Health. 2025 Apr 29;22(5):704. doi: 10.3390/ijerph22050704 (PMC12110824; doi:10.3390/ijerph22050704)
Supplement: Supplementary file 1 [file ijerph-22-00704-s001.zip › ijerph-3558133-supplementary.pdf]

Supplemental Materials

I. **Supplemental Methods**

II. **Supplemental Tables**

III. **Supplemental Figures**

I. Supplemental Methods

Supplemental details for the Cognitive Abilities Screening Instrument (CASI) bifactor score

The Cognitive Abilities Screening Instrument (CASI) in the Adult Changes in Thought (ACT) study is an instrument that measures global cognition. More details on the CASI instrument are available on the ACT study website: https://www.actagingresearch.org/index.php/resources/act-data-repository/act-study-visit-data/biennial-study-visit-data/form84. An item response theory (IRT) model was used to create the CASI bifactor score from its 39 items. In IRT, two sets of parameters are computed for each binary item, *difficulty* (the level of cognitive ability needed to answer correctly) and *discrimination* (how well the item distinguishes between high and low cognitive ability). The graded response model extends this with additional difficulty thresholds for polytomous items. The model was fit using the lavaan package in R which assigned individuals an overall score (doi:10.18637/jss.v048.i02). The lavaan package in R limits ordinal variables to 10 categories, so we combined some response levels for animals and draw, using the same groupings as in Mukherjee et al. 2023. Other rare responses for items “When Were You Born—Year”, “Identify Part of Body”, and “Judgement” were combined. We also combined the object naming items into one item, both correct vs not. R code for the CASI bifactor score is available from the corresponding author upon request.

Supplemental Methods S1 shows elements of the fitted IRT model. So that we can discuss the scores in meaningful units, we **standardized** the scores to a mean of 0 and SD of 1 for the initial (1992-1994) ACT cohort at baseline by subtracting 0.0567 and then dividing by 0.7924.

Supplemental Method S1. CASI bifactor model. All items are a reflection of the Cognitive Abilities Screening Instrument (CASI) assessing global cognition. There is a methods effects factor, F1, for the recall items. (Variables not listed in the order the items are administered in; recall items were rearranged so that F1 was easier to read).

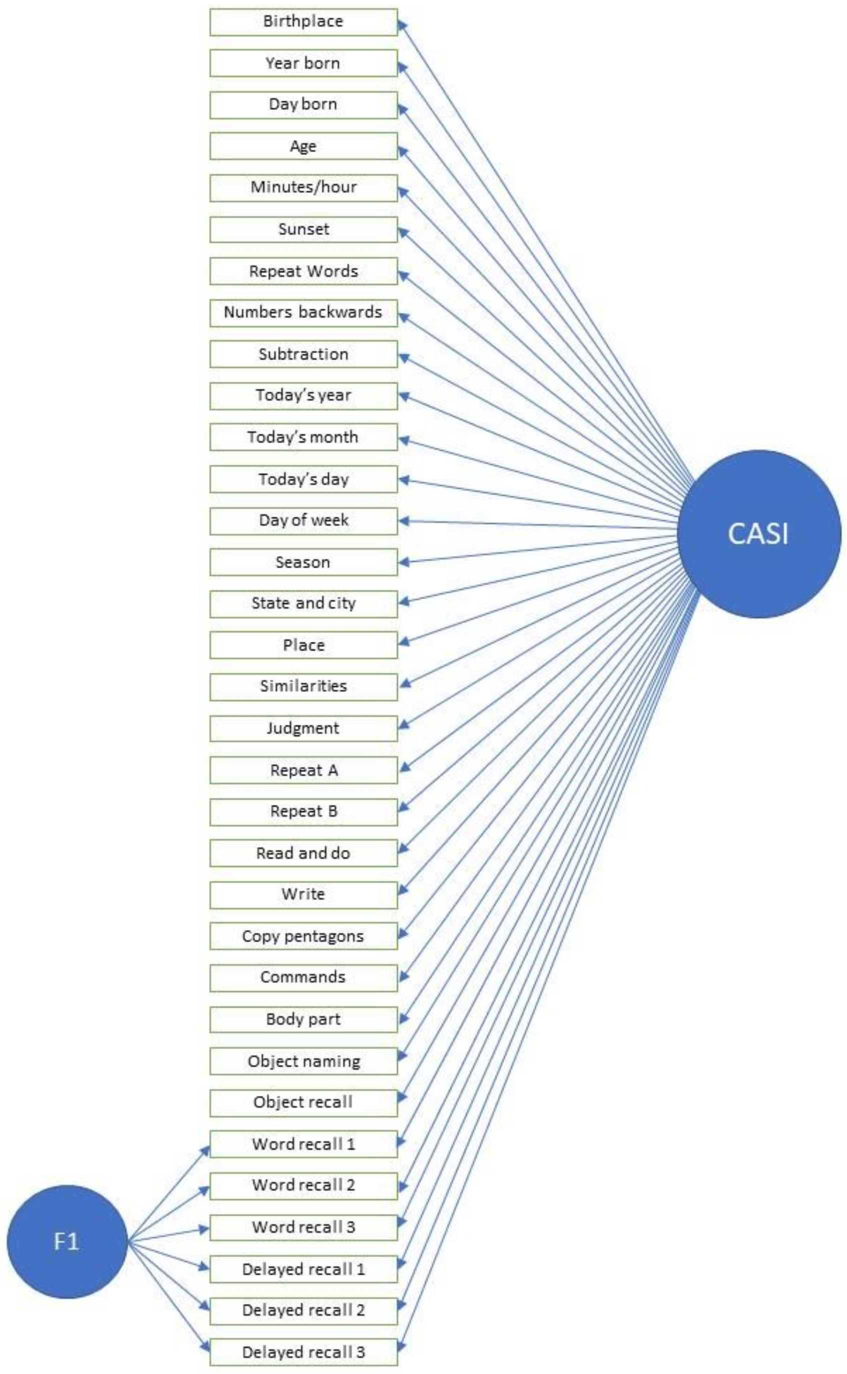

II. Supplemental Tables

Table S1. Characteristics of participants in the Activity Monitor ACT study that were excluded versus included in the analytic cohort (N=1885).

	**Excluded from Analytical Cohort**	**Included from Analytical Cohort**
	**N = 980** [a]	**N = 905** [a]
**Age (Years) Category,** n (%)		
65-74	264 (26.9%)	378 (41.8%)
74-84	374 (38.2%)	381 (42.1%)
85+	342 (34.9%)	146 (16.1%)
**Age (Years),** Mean (SD)	81.3 (8.1)	77.6 (6.9)
**Gender,** n (%)		
Women	592 (60.4%)	502 (55.5%)
Men	388 (39.6%)	403 (44.5%)
**Race,** n (%)		
Asian	43 (4.4%)	28 (3.1%)
Black	29 (3.0%)	13 (1.4%)
White	853 (87.0%)	820 (90.6%)
Other or mixed [b]	50 (5.1%)	43 (4.8%)
**Latino/Hispanic Ethnicity**, n (%)	20 (2.0%)	12 (1.3%)
**Currently Work for Pay,** n (%)	117 (11.9%)	168 (18.6%)
**Education Level 16+ years,** n (%)	646 (65.9%)	677 (74.8%)
**Live alone**, n (%)	421 (43.0%)	308 (34.0%)

[a] Percentage is calculated out of the total N=980 (left column) and 905 (right column)**.** The following variables had missing data for those excluded: Race n=5, Latino/Hispanic Ethnicity n=4, Work status n=3, Live arrangement n=3 and for those included: : Race n=1, Latino/Hispanic Ethnicity n=3.

[b] Native Hawaiian/Pacific Islander/American Indian/Alaskan Native/Mixed/Other

Table S2. Demographic characteristics at device wear for the Adult Changes in Thought (ACT) study analytic cohort (N = 905).

	**Activity Monitor ACT Cohort**	**ADL: 0**	**ADL: 1+**
	**N = 905** [a]	**N = 425** [a]	**N = 480** [a]
**Age (Years) Category,** n (%)			
65-74	378 (41.8%)	239 (56.2%)	139 (29.0%)
74-84	381 (42.1%)	156 (36.7%)	225 (46.9%)
85+	146 (16.1%)	30 (7.1%)	116 (24.2%)
**Age (Years),** Mean (SD)	77.6 (6.9)	75.3 (5.67)	79.7 (7.22)
**Gender,** n (%)			
Women	502 (55.5%)	223 (52.5%)	279 (58.1%)
Men	403 (44.5%)	202 (47.5%)	201 (41.9%)
**Race,** n (%)			
Asian	28 (3.1%)	12 (2.8%)	16 (3.3%)
Black	13 (1.4%)	5 (1.2%)	8 (1.7%)
White	820 (90.6%)	394 (92.7%)	426 (88.8%)
Other or mixed[+]	43 (4.8%)	14 (3.3%)	30 (6.3%)
**Latino/Hispanic Ethnicity**, n (%)	12 (1.3%)	5 (1.2%)	7 (1.5%)
**Currently Work for Pay,** n (%)	168 (18.6%)	100 (23.5%)	68 (14.2%)
**Education Level 16+ years,** n (%)	677 (74.8%)	346 (81.4%)	331 (69.0%)
**Live alone**, n (%)	308 (34.0%)	124 (29.2%)	184 (38.3%)

[a] Percentage is calculated out of the total N=905 (All), N=425 (ADL=0), N=480 (ADL>0). Missing values of ADL at baseline (n=14) were imputed, for purposes of this summary table, using the most recent non-missing value within the 10-year window prior to baseline. The following variables had missing data: Race n=1, Latino/Hispanic Ethnicity n=3

[+] Native Hawaiian/Pacific Islander/Mixed or Other

Table S3. Table of comorbidities and clinical characteristics at device wear for the Adult Changes in Thought (ACT) study analytical cohort (N=905).

	**ALL N = 905** [a]	**ADL: 0 N = 425** [a]	**ADL: 1+ N = 480** [a]
	**N (%) or Median [Q1, Q3]**	**N (%) or Median [Q1, Q3]**	**N (%) or Median [Q1, Q3]**
**Self-rated health,** n (%)			
Excellent	178 (19.7%)	128 (30.1%)	50 (10.4%)
Very good	393 (43.4%)	209 (49.2%)	184 (38.3%)
Good	271 (29.9%)	76 (17.9%)	195 (40.6%)
Fair/Poor	63 (7.0%)	12 (2.8%)	51 (10.6%)
**Depressive symptoms CES-D Score ≥ 10,** n (%)	77 (8.5%)	19 (4.5%)	58 (12.1%)
**Depressive symptoms (CES-D)**	2 [1, 5]	2 [0, 4]	3 [1, 6]
**Charlson Comorbidity Index**	0 [0, 2]	0 [0, 1]	1 [0, 2]
**Activity of Daily Living (ADL) score**	1 [0, 2]	0 [0, 0]	2 [1, 4]
**Short Performance-Based Physical Function (sPPF) score**	9 [7, 11]	10.0 [9, 11]	8 [6, 10]
**CASI score,** Mean (SD)	0.6 (1.0)	0.7 (0.9)	0.5 (1)
**BMI**, Mean (SD)	26.9 (4.8)	26.2 (4.2)	27.5 (5.1)

Note. Abbreviations: ADL: Activities of Daily Living; BMI, body mass index; CES-D: total score on the shortened 10-item Center for Epidemiology Studies Depression Scale, CASI: standardized CASI score which was computed from summing each of the nine CASI domain scores; sPPF: Short Performance-Based Physical Function .

[a] Percentage is calculated out of the total N=905, which includes those with missing data in**.** Missing values of ADL at baseline were imputed, for purposes of this summary table, using the most recent non-missing value within the 10-year window prior to baseline. The following variables had missing data: Charlson Comorbidity Index (n=40), Pain index (n=65), ADL score (n=13), sPPF (n=38)

Table S4. Associated change in PA, SB, and sleep behavior outcomes for a 1-unit decrease in the intercept (baseline) and 0.3-unit decrease in slope of the Short Performance-Based Physical Function Score (sPPF) score trajectory (N=905)

**Outcome**	**Individual Trajectory Features**	**Est** [a]	**Bootstrap 95% CI** [b]	**95% CI** [c]
**AP sit time (minutes/day)** †	Intercept (baseline)	6.6	(1.2, 11.7)	(1.7, 11.5)
	Slope	6.6	(-32.1, 52.0)	(-25.2, 38.4)
**AP stand time (minutes/day)** †	Intercept (baseline)	-2.5	(-7.0, 2.0)	(-6.6, 1.7)
	Slope	5.3	(-29.1, 38.7)	(-21.8, 32.3)
**AP step time (minutes/day)** †	Intercept (baseline)	-4.1	(-5.7, -2.6)	(-5.6, -2.6)
	Slope	-11.8	(-29.7, 0.1)	(-21.7, -2)
**AP num steps (count/day)** †	Intercept (baseline)	-359	(-502, -226)	(-494, -225)
	Slope	-1180	(-2853, -185)	(-2063, -297)
**AP bout duration (minutes/day)** †	Intercept (baseline)	0.4	(0, 0.8)	(0.1, 0.8)
	Slope	0.2	(-3.4, 3.3)	(-1.9, 2.4)
**AG light activity (minutes/day)** ‡	Intercept (baseline)	-1.6	(-4.9, 1.6)	(-4.6, 1.4)
	Slope	-2.1	(-26.3, 21.9)	(-21.7, 17.6)
**AG moderate to vigorous activity (minutes/day)** ‡	Intercept (baseline)	-4.4	(-6.1, -3.0)	(-6.0, -2.7)
	Slope	-15.7	(-35.6, -2.3)	(-26.5, -5.0)
**Time-in-bed (minutes/day)**	Intercept (baseline)	1.1	(-2.1, 4.3)	(-1.9, 4.0)
	Slope	6.0	(-15.0, 30.3)	(-13.1, 25.2)
**PROMIS Sleep Disturbance Score**^	Intercept (baseline)	0.2	(-0.2, 0.5)	(-0.2, 0.5)
	Slope	1.6	(-0.6, 4.4)	(-0.7, 3.8)

Note. Abbreviations: AP: activPAL, AG: ActiGraph

[a] Fitted with the linear mixed effects model adjusted for age in years, gender, body mass index kg/m^2, education (16+ years versus <16 years), current work for pay, CESD Score, CASI IRT, live alone vs with others, self-rated health (4-level, Excellent/Very good/Good vs Fair/Poor).

[b] 95% Confidence interval from percentile bootstrap algorithm with 1000 bootstrap replications.

[c] 95% Confidence intervals based on the naïve model-based standard errors that ignore the uncertainty in the estimated intercept and slope (BLUPS).

^ PROMIS Sleep Disturbance Score has missing value n = 64.

† Additionally adjusted for AP weartime

‡ Additionally adjusted for AG weartime

Table S5. Inverse-probability weighted (IPW) analysis for the associated change in mean PA, SB, and sleep behavior outcomes for a 1-unit decrease in the intercept (baseline) and 0.3-unit decrease in slope of the Short Performance-Based Physical Function Score (sPPF) score trajectory (N=905).

Outcome		Est*	Bootstrap 95% CI**
**AP sit time (minutes/day)** †	Intercept (baseline)	8.6	(2.5, 14.5)
	Slope	14.7	(-31.6, 57.4)
**AP stand time (minutes/day)** †	Intercept (baseline)	-4.6	(-9.7, 0.9)
	Slope	-4	(-37, 39.9)
**AP step time (minutes/day)** †	Intercept (baseline)	-4	(-5.4, -2.6)
	Slope	-10.7	(-29.2, 0.8)
**AP num steps (count/day)** †	Intercept (baseline)	-353	(-470, -227)
	Slope	-1026	(-2804, -66.8)
**AP bout duration (minutes/day)** †	Intercept (baseline)	0.5	(-0.04, 1)
	Slope	1	(-3.2, 4.5)
**AG light activity (minutes/day)** ‡	Intercept (baseline)	-2.2	(-5.8, 1.9)
	Slope	-1	(-25.7, 22.8)
**AG moderate to vigorous activity (minutes/day)** ‡	Intercept (baseline)	-4.1	(-5.4, -2.7)
	Slope	-13	(-35.2, 0.2)
**Time-in-bed (minutes/day)**	Intercept (baseline)	0.3	(-3.2, 4)
	Slope	16.7	(-13.4, 46.3)
**PROMIS Sleep Disturbance Score**^	Intercept (baseline)	0.03	(-0.4, 0.5)
	Slope	1.8	(-1, 4.4)

Note. Abbreviations: AP: activPAL, AG: ActiGraph

* Estimated from the regression coefficient from the IPW multivariable linear regression model. The intercept (baseline) and slope exposures were derived from with the linear mixed effects (lme) model fit to the longitudinal data for ADL collected during the10 years prior to device wear and included in separate outcome models. The outcome and lme models were also adjusted for age in years, gender, body mass index kg/m^2, education (16+ years versus <16 years), current work for pay, CESD Score, CASI IRT, live alone vs with others, self-rated health (4-level, Excellent/Very good/Good vs Fair/Poor). The inverse probability weights were derived from the fitted logistic regression model to derive the probability of having complete data (n=905), and included as predictors: ability to walk a half mile, sPPF, age, gender, 16+ years education (yes/no), living alone, current work for pay (yes/no), self-rated health (Excellent, very good, good, fair/poor), CASI IRT; this IPW model included 1857 individuals (excluding 28 of 1885 invited who were missing some baseline exposures).

**: Confidence interval from percentile bootstrap algorithm with 1000 bootstrap replications.

***: PROMIS Sleep Disturbance Score has missing value n = 64.

† Additionally adjusted for AP weartime

‡ Additionally adjusted for AG weartime

Table S6. Associated difference in PA, SB, and sleep behavior outcomes for a 1-unit increase in the intercept (baseline) and 0.4-unit increase in the slope of the Activity of Daily Living (ADL) score trajectory (N=905) **(higher scores indicate more limitation)**

**Outcome**	**Individual Trajectory Features**	**Est [a]**	**Bootstrap 95% CI [b]**	**95% CI [c]**
**AP sit time (min/day) †**	Intercept (baseline)	8.8	(2.9, 13.9)	(3.8, 13.7)
	Slope	35.0	(4.3, 65.0)	(9.4, 60.6)
**AP stand time (min/day) †**	Intercept (baseline)	-5.1	(-9.2, 0)	(-9.3, -0.8)
	Slope	-20.6	(-43.1, 4.4)	(-42.5, 1.2)
**AP step time (min/day) †**	Intercept (baseline)	-3.7	(-5.2, -2.2)	(-5.2, -2.2)
	Slope	-14.4	(-24.2, -5.6)	(-22.3, -6.5)
**AP num steps (count/day) †**	Intercept (baseline)	-348	(-478, -225)	(-486, -210)
	Slope	-1372	(-2223, -638)	(-2083, -661)
**AP bout duration (min/day) †**	Intercept (baseline)	0.8	(0.3, 1.3)	(0.5, 1.1)
	Slope	3.5	(0.8, 6.2)	(1.7, 5.2)
**AG light activity (min/day) ‡**	Intercept (baseline)	-0.9	(-4.1, 2.7)	(-4.0, 2.2)
	Slope	-8.0	(-23.9, 9.8)	(-23.8, 7.9)
**AG moderate to vigorous activity (min/day) ‡**	Intercept (baseline)	-3.3	(-5.0, -1.7)	(-5.0, -1.6)
	Slope	-13.0	(-22.6, -5.0)	(-21.7, -4.3)
**Time-in-bed (min/day)**	Intercept (baseline)	4.5	(1.0, 8.2)	(1.5, 7.5)
	Slope	25.5	(6.5, 43.5)	(10.2, 40.8)
**PROMIS Sleep Disturbance Score^**	Intercept (baseline)	0.2	(-0.1, 0.6)	(-0.1, 0.6)
	Slope	1.0	(-0.9, 3.1)	(-0.9, 2.9)

Note. Abbreviations: AP: activPAL, AG: ActiGraph

[a] Fitted with the linear mixed effects model adjusted for age in years, gender, body mass index kg/m^2, education (16+ years versus <16 years), current work for pay, CESD Score, CASI IRT, live alone vs with others, self-rated health (4-level, Excellent/Very good/Good vs Fair/Poor).

[b] 95% Confidence interval from percentile bootstrap algorithm with 1000 bootstrap replications.

[c] 95% Confidence intervals based on the naïve model-based standard errors that ignore the uncertainty in the estimated intercept and slope (BLUPS).

† Additionally adjusted for AP weartime

‡ Additionally adjusted for AG weartime

^ PROMIS Sleep Disturbance Score has missing value n = 64.

Table S7. Inverse-probability weighted (IPW) analysis associated difference in PA, SB, and sleep behavior outcomes for a 1-unit increase in the intercept (baseline) and 0.4-unit increase in the slope of the Activity of Daily Living (ADL) score trajectory (N=905)**.**

**Outcome**		**Est***	**Bootstrap 95% CI****
**AP sit time (minutes/day)** †	Intercept (baseline)	12.4	(5.6,18.5)
	Slope	47.2	(12.6, 85.9)
**AP stand time (minutes/day)** †	Intercept (baseline)	-8.4	(-13.8, -2.3)
	Slope	-31.6	(-63.7, -2.6)
**AP step time (minutes/day)** †	Intercept (baseline)	-4.1	(-5.5, -2.5)
	Slope	-15.6	(-24.7, -7.1)
**AP num steps (count/day)** †	Intercept (baseline)	-371	(-491, -254)
	Slope	-1447	(-2255, -763)
**AP bout duration (minutes/day)** †	Intercept (baseline)	1	(0.4, 1.7)
	Slope	4.3	(1.1, 8.0)
**AG light activity (minutes/day)** ‡	Intercept (baseline)	-2.1	(-5.7, 2.3)
	Slope	-11.9	(-31.7, 8.0)
**AG moderate to vigorous activity (minutes/day)** ‡	Intercept (baseline)	-3.2	(-4.6, -1.9)
	Slope	-13.2	(-21.5, -6.0)
**Time-in-bed (minutes/day)**	Intercept (baseline)	5.2	(0.9, 9.2)
	Slope	31.6	(9.5, 51.8)
**PROMIS Sleep Disturbance Score**^	Intercept (baseline)	0.2	(-0.3, 0.6)
	Slope	0.3	(-2.1, 2.7)

Note. Abbreviations: AP: activPAL, AG: ActiGraph

* Estimated from the regression coefficient from the IPW multivariable linear regression model. The intercept (baseline) and slope exposures were derived from with the linear mixed effects (lme) model fit to the longitudinal data for ADL collected during the10 years prior to device wear and included in separate outcome models. The outcome and lme models were also adjusted for age in years, gender, body mass index kg/m^2, education (16+ years versus <16 years), current work for pay, CESD Score, CASI IRT, live alone vs with others, self-rated health (4-level, Excellent/Very good/Good vs Fair/Poor). The inverse probability weights were derived from the fitted logistic regression model to derive the probability of having complete data (n=905), and included as predictors: ability to walk a half mile, sPPF, age, gender, 16+ years education (yes/no), living alone, current work for pay (yes/no), self-rated health (Excellent, very good, good, fair/poor), CASI IRT; this IPW model included 1857 individuals (excluding 28 of 1885 invited who were missing some baseline exposures).

**: Confidence interval from percentile bootstrap algorithm with 1000 bootstrap replications.

***: PROMIS Sleep Disturbance Score has missing value n = 64.

† Additionally adjusted for AP weartime

‡ Additionally adjusted for AG weartime

III. Supplemental Figures

Supplemental Figure S1. Strobe diagram for inclusion in analytic sample.

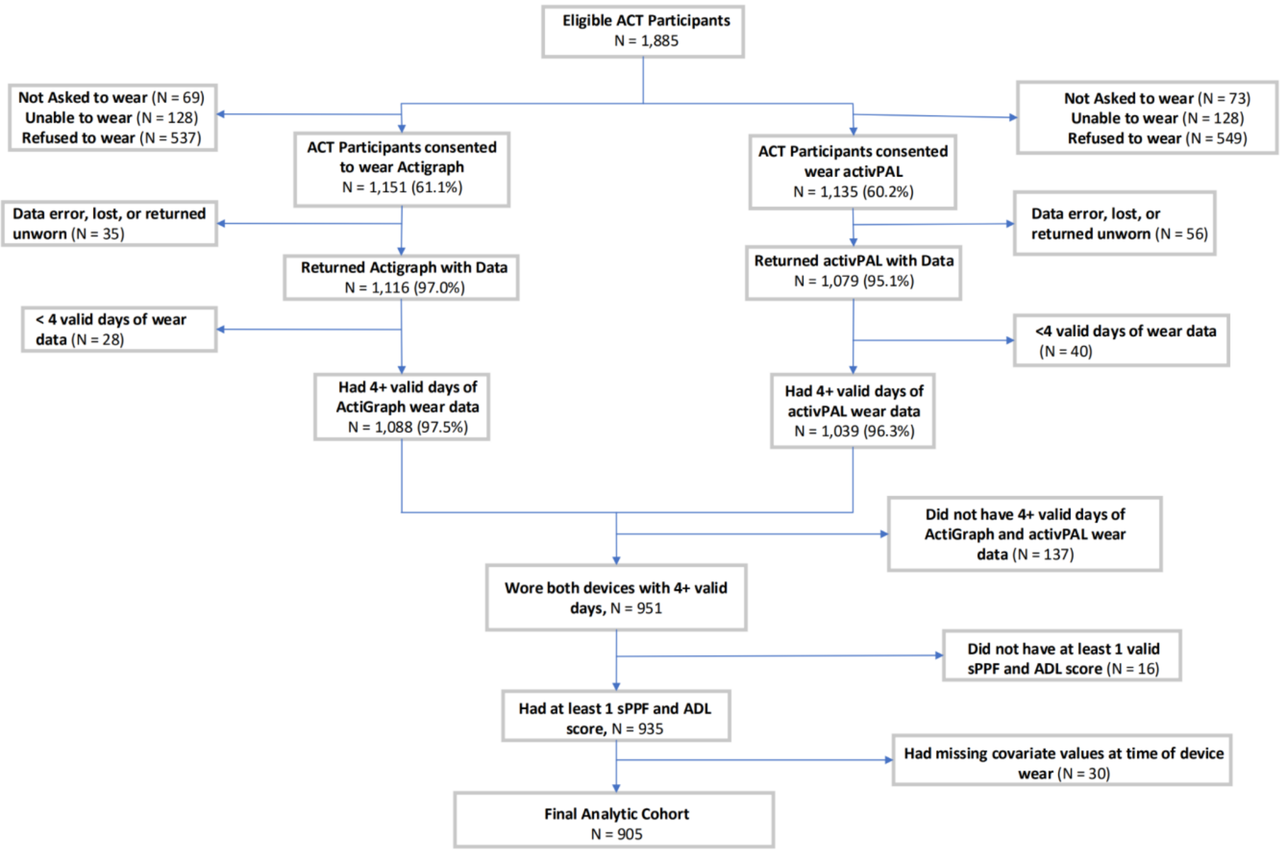

Figure S2. Individual trajectories for the activities for the Activities of Daily Living (ADL) and short version of the Performance-Based Physical Function (sPPF) scores for 20 randomly selected subjects in Adult Changes in Thought (ACT) Study with at least 2 observations for each measure.

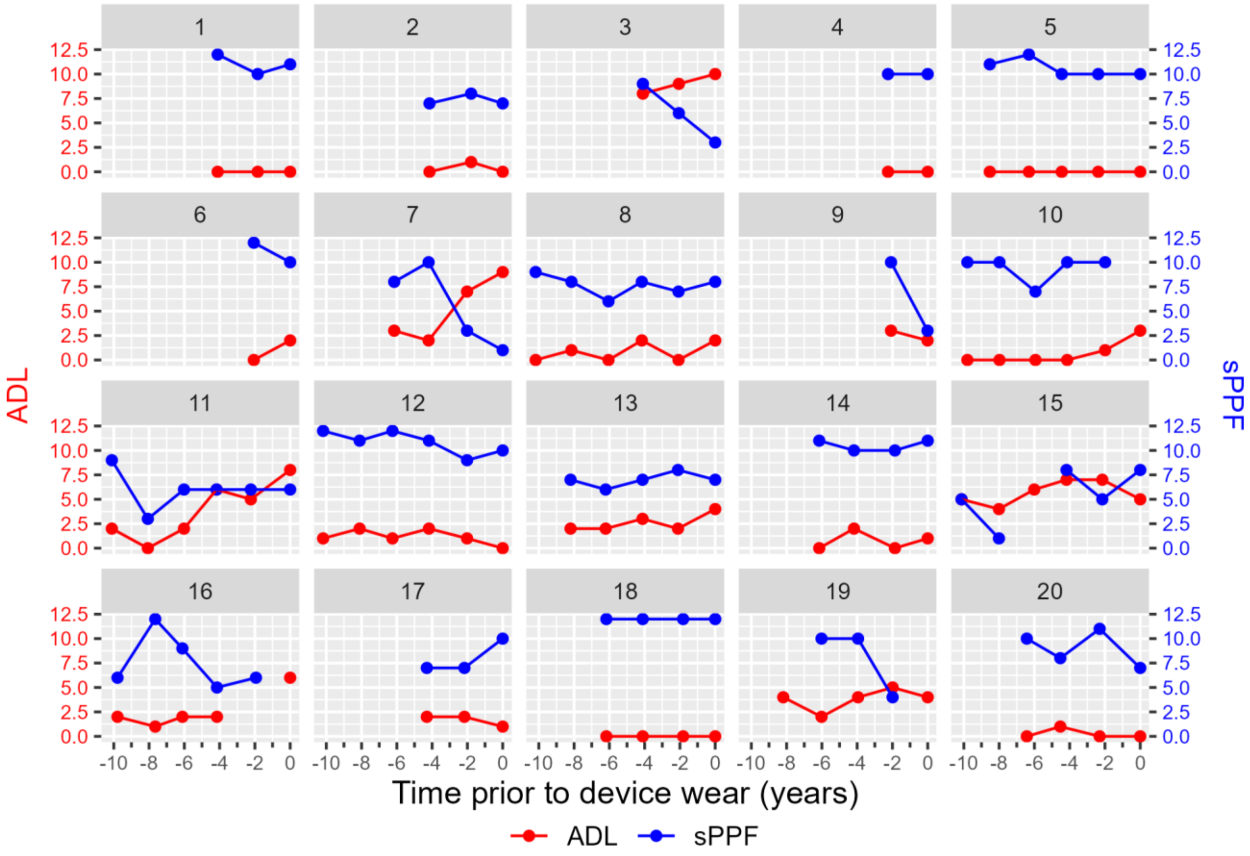

Figure S3. Unadjusted association between sPPF (top) and ADL (bottom) intercepts with physical behavior outcomes of interest [a].

A) sPPF

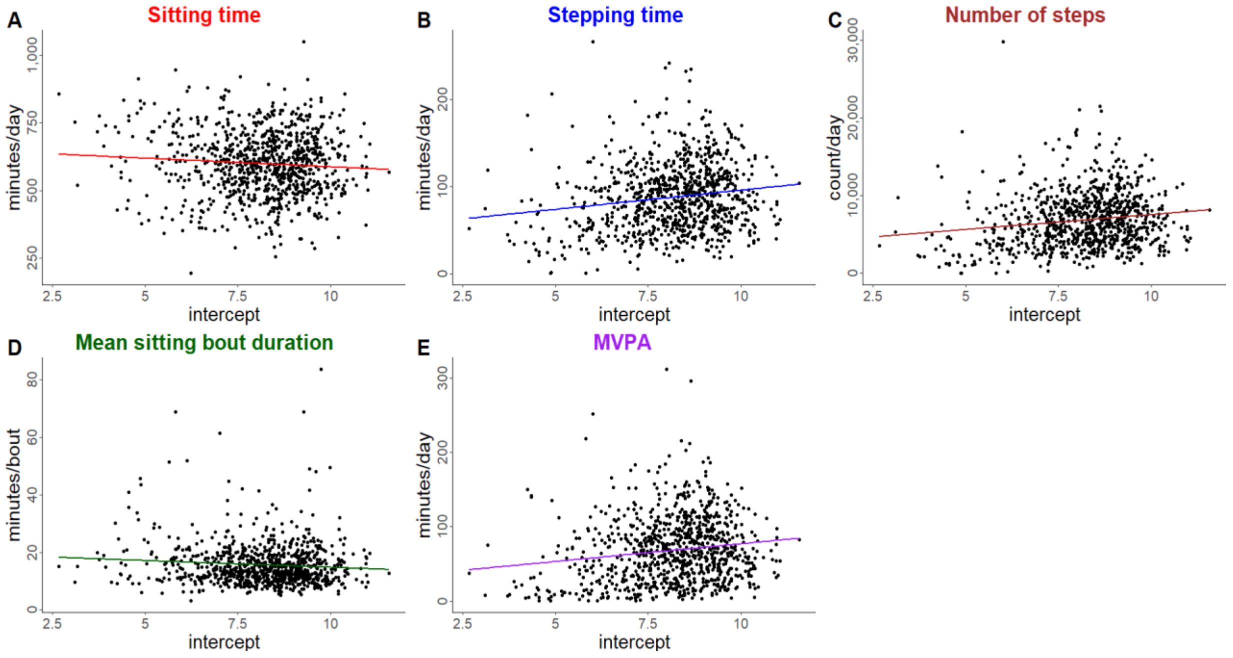

B) ADL

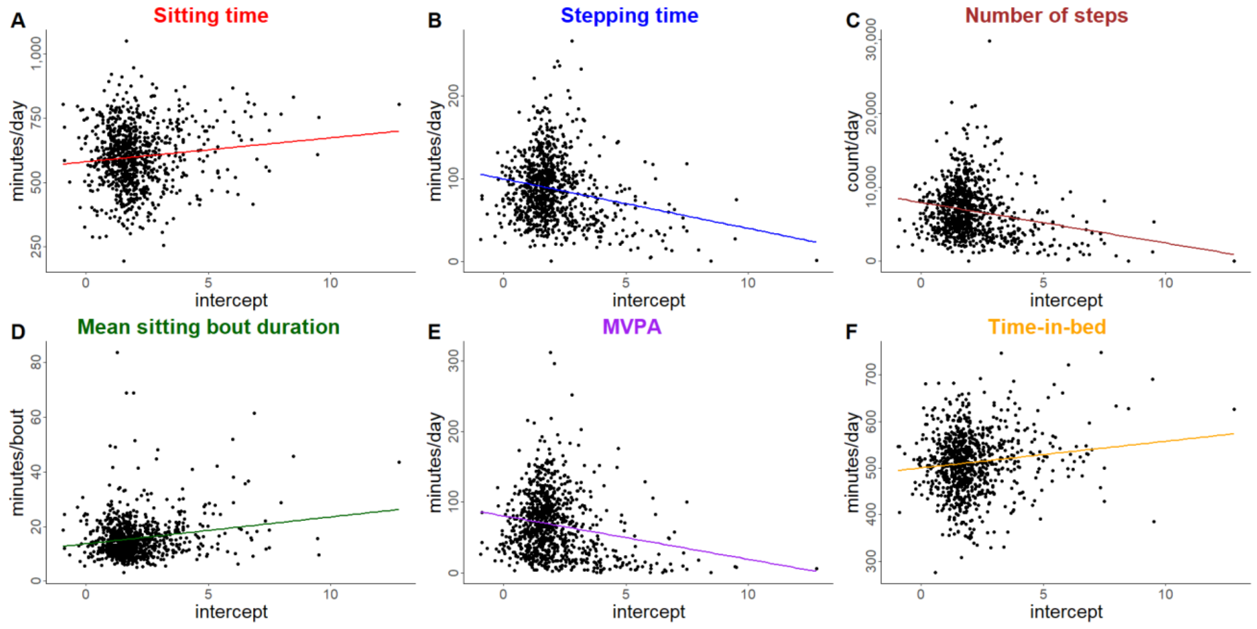

[a] Only outcomes with a statistically significant association with the physical function trajectory in adjusted models are displayed.
